# Ovarian Cancer Incidence in the U.S. and Toxic Emissions from Pulp and Paper Plants: A Geospatial Analysis

**DOI:** 10.3390/ijerph15081619

**Published:** 2018-07-31

**Authors:** Carol Hanchette, Charlie H. Zhang, Gary G. Schwartz

**Affiliations:** 1 Department of Geography and Geosciences, University of Louisville, 2301 S 3rd Street, Louisville, KY 40292-0001, USA; 2 Department of Population Health, School of Medicine & Health Sciences, University of North Dakota, 1301 N Columbia Rd., Stop 9037, Grand Forks, ND 58202-9037, USA; gary.schwartz@med.und.edu

**Keywords:** ovarian cancer incidence, whites, toxic emissions, pulp and paper, geospatial analysis

## Abstract

Ovarian cancer is the fifth leading cause of female cancer mortality in the U.S. and accounts for five percent of all cancer deaths among women. No environmental risk factors for ovarian cancer have been confirmed. We previously reported that ovarian cancer incidence rates at the state level were significantly correlated with the extent of pulp and paper manufacturing. We evaluated that association using county-level data and advanced geospatial methods. Specifically, we investigated the relationship of spatial patterns of ovarian cancer incidence rates with toxic emissions from pulp and paper facilities using data from the Environmental Protection Agency’s Toxic Release Inventory (TRI). Geospatial analysis identified clusters of counties with high ovarian cancer incidence rates in south-central Iowa, Wisconsin, New York, Pennsylvania, Alabama, and Georgia. A bivariate local indicator of spatial autocorrelation (LISA) analysis confirmed that counties with high ovarian cancer rates were associated with counties with large numbers of pulp and paper mills. Regression analysis of state level data indicated a positive correlation between ovarian cancer and water pollutant emissions. A similar relationship was identified from the analysis of county-level data. These data support a possible role of water-borne pollutants from pulp and paper mills in the etiology of ovarian cancer.

## 1. Introduction

Ovarian cancer is the fifth leading cause of female cancer mortality and accounts for five percent of all cancer deaths among women in the U.S. The estimated number of new and fatal ovarian cancers in the U.S. in 2017 are 22,440 and 14,080, respectively [1]. Although five-year survival rates for ovarian cancer have shown improvement over the past four decades, the five-year survival rate is only 47 percent for all stages combined and is 29 percent for late-stage (metastatic) cancers, where the majority of ovarian cancers are diagnosed. Presently, there is no accurate modality or biomarker for early diagnosis [1]. The latency period for ovarian cancer (i.e., the time from inception of disease to clinical presentation) is unknown, but is estimated at 15–20 years [2].

The best-known risk factor for ovarian cancer is family history, notably among women with BCRA1/BRCA2 mutations. However, this factor explains only about 10 percent of all cases [3]. Other known risk factors are reproductive: advanced age, nulliparity, and infertility. There is a protective effect for breastfeeding and other hormonal factors, such as early age at menarche and/or late age at menopause, that are associated with a reduced number of ovulatory cycles. Obesity, endometriosis and pelvic inflammatory disease also are associated with increased risk [4,5]. However, no consistent significant environmental risk factors for ovarian cancer have been identified, although the use of talc (e.g., in talcum powder) has received mixed support [6,7].

Hanchette and Schwartz’s publication concerning a protective effect of vitamin D on mortality from prostate cancer [8,9] stimulated investigations into the possible protective effect of Vitamin D/sunlight in several cancers, including ovarian cancer [10,11,12,13,14]. Although an inverse correlation between ovarian cancer and ultraviolet radiation was reported in ecological studies, a systematic review of the analytic epidemiologic literature (i.e., case-control and cohort studies) does not support a protective effect of Vitamin D in ovarian cancer [15].

There are marked differences in the geographic distribution of ovarian cancer incidence and mortality, both nationally and worldwide [14,16,17,18,19]. Few studies have attempted to explain these spatial patterns. In general, higher incidence and mortality rates are found at higher latitudes. In 2012, mortality rates per 100,000 were 5.19 for the European Union (EU) and 4.85 for the U.S., which ranked 26th [16]. From 1970 to 2010, mortality rates have declined overall and by histologic subtype in the E.U. (10 percent) and U.S. (16 percent), a finding that has been partially attributed to the use of oral contraceptives [16,20].

The age-adjusted ovarian cancer incidence rate in the U.S. (for all race/ethnicities) for the years 2010–2014 was 11.4 per 100,000 and ranged from 9.7 in Louisiana, Hawaii, and Mississippi to 13.0 in West Virginia [21]. With the exception of West Virginia, all states with incidence rates in the highest quartile are in the northern U.S. Incidence rates for white women are considerably higher (11.8) than those for African-American women (9.2). Ovarian cancer mortality rates for the same time period ranged from 8.9 in Oregon to 5.3 per 100,000 in North Dakota. 

Some of the variation in the geographic distribution of ovarian cancer rates may be related to levels of industrialization. Ovarian cancer has been associated with industrial pollution in ecological studies [22], and there is evidence that occupational exposure among pulp and paper mill employees increases the risk [23,24]. A Norwegian study found a (not significantly elevated) odds ratio of 2.02 for exposure to asbestos [25]. This was supported by a recent meta-analysis of ovarian cancer and asbestos [26]. In the U.S., a positive correlation between state-level ovarian cancer incidence rates and pulp and paper manufacturing has been reported by Schwartz and Sahmoun (2014) [27]. A similar relationship was reported for geographic regions in Spain [22].

Although examining relationships between disease rates and environmental factors using state-level data is often a valuable first step in hypothesis development, an analysis of data at finer geographical scales (e.g., counties) can provide a more in-depth understanding of spatial patterns. This is because associations between disease rates and environmental factors are sensitive to the choice of the geographic scale to which the data have been aggregated, a spatial issue known as the modifiable areal unit problem (MAUP) [28,29,30]. Here, we applied advanced geospatial methods to the examination of county-level data on ovarian cancer incidence and Toxic Release Inventory (TRI) emissions data in order to gain insight into the spatial distribution of ovarian cancer and pulp and paper manufacturing. We report statistically significant associations between ovarian cancer incidence rates in the U.S. and toxic surface water emissions from pulp and paper plants.

## 2. Materials and Methods

### 2.1. Data on Ovarian Cancer and Toxic Emissions

We obtained age-standardized 2009–2013 county-level ovarian cancer incidence rates for white women from State Cancer Profiles, a collaboration of the National Cancer Institute and the Centers for Disease Control and Prevention (CDC) [31]). For comparison, we obtained state-level data for the same time period. Because African-American women have lower ovarian cancer incidence rates than whites [32,33], our analyses are restricted to whites. Because rates for non-Hispanic whites were not uniformly available, we used data on all whites.

**Figure 1:**
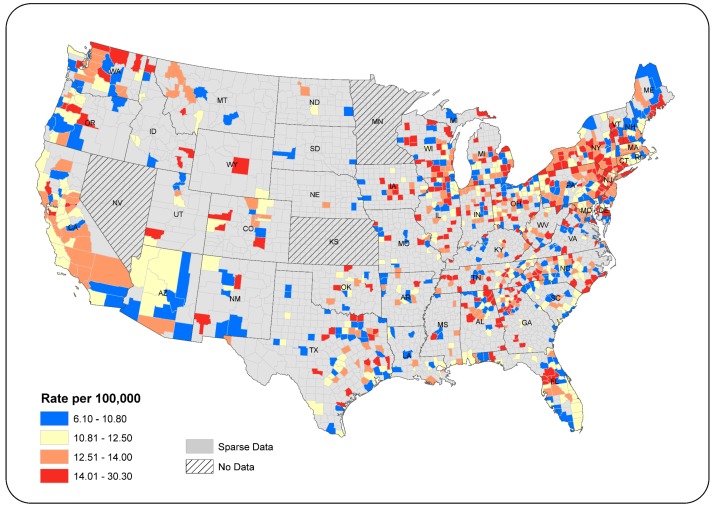
County-level ovarian cancer incidence per 100,000 population, 2009–2013, whites.

State-level data on incidence of ovarian cancer included all states in the conterminous U.S., excluding Nevada (which did not report data to CDC). County-level data for the states of Nevada, Kansas, and Minnesota were not available. Cancer incidence rates were suppressed for many counties due to sparse numbers (i.e., less than 16 records, a standard set by the CDC). Therefore, our analysis of ovarian cancer incidence for white females in the U.S. was restricted to 987 counties across 45 states and Washington D.C. (Figure 1).

We obtained data on toxic air and water releases from pulp and paper mills (NAICS Code 322) from the Environmental Protection Agency’s Toxic Release Inventory (TRI) for the time period 1988–2012 [34]. Due to the potential long latency period of ovarian cancer, we examined toxic air and water releases for a 25-year time period. Included in these data were number of facilities and amount and type of discharge by ZIP code, county, and EPA region. Data were also available for individual facilities (i.e., points on a map), enabling a finer level of geographic precision. Using each site’s latitude/longitude coordinates, we mapped locations of individual facilities and overlaid them with ovarian cancer incidence rates that are aggregated to state and county boundaries. We also downloaded 2000–2012 data for two subcategories of pulp and paper mill emissions: Occupational Safety and Health Administration (OSHA) carcinogens and dioxin; the latter is used in the chlorine-bleaching process of paper production [26,35]. Since 2000, separate data on these chemicals can be downloaded from EPA’s website.

### 2.2. Exploratory Spatial Data Analysis Methods

We first performed exploratory spatial data analysis (ESDA) to examine the geographic distributions of ovarian cancer incidence and paper and pulp facilities and to gauge whether these two variables are spatially related to each other [36]. Among various ESDA techniques, the global Moran’s I allows a diagnosis of the overall spatial distribution of ovarian cancer incidence across the U.S. (i.e., clustered, dispersed, or random), whereas the local indicator of spatial autocorrelation (LISA) identifies local clusters or outliers that were comprised by counties having similar or dissimilar rates of incidence with their neighbors. Furthermore, a bivariate LISA analysis allows us to examine whether counties with high rates of ovarian cancer incidence were spatially associated with neighboring counties that had large numbers of paper mills, or vice versa [37,38]. All analyses were conducted using Environmental Systems Research Institute (ESRI) ArcGIS 10.5 and GeoDa, an open source software for spatial statistical analysis developed by Anselin and colleagues [39].

### 2.3. Regression Analysis of the Relationship between Paper Mill Emissions and Ovarian Cancer

To permit comparison with prior research that reported positive associations between ovarian cancer incidence and paper manufacturing, we first conducted regression analyses using state-level data. Ovarian cancer incidence rates were the dependent variable; air and water emissions were the independent variables. These procedures were conducted for: (1) all emissions, 1988–2012; (2) dioxin, 2000–2012; and (3) OSHA carcinogens, 2000–2012, respectively (Table 1).

Geographic research has suggested that issues including spatial autocorrelation and spatially varying relationships can bias the estimates of model parameters in regression analyses when geographically referenced data are used [39,40,41]. Therefore, alternative spatial regression models (i.e., lag or error) could be used to address the spatial autocorrelation problem if statistically significant Moran’s I is being flagged. Likewise, geographically weighted regression (GWR) is often employed to generate local regression models that address inconsistent relationships between dependent and independent variables across a study area [41,42]. Therefore, in order to investigate whether ovarian incidence rates were related to TRI emissions from pulp and paper plants, we used ordinary least squares (OLS) regression first for both the state- and county-level data. We next performed spatial lag models for the state-level data in light of the statistically significant spatial dependence across the 45 states. For the county-level data, we performed GWR models in consideration of the large sample size (i.e., *n* = 987) and possible regional heterogeneities in factors that potentially affect the incidence of ovarian cancer incidence across U.S. counties.

## 3. Results

### 3.1. Spatial Distribution of Ovarian Incidence Rates

The county-level age-standardized ovarian cancer incidence rates (2009–2013) varied from 6.1 to 30.3 per 100,000 (Figure 1), a range considerably larger than that for the state-level data (i.e., 9.6–13.4 for the same time period as displayed in Figure 3). Incidence rates were classified using the quantile method [43]. Rates above the 50th percentile are shown in orange and red. Representation in counties in the West can be misleading because of the large geographic size of these counties. Areas with higher rates are located in Washington, Wisconsin, New York, and New Jersey, with additional foci of high rates in northeastern Alabama, western Florida, and coastal North Carolina.

**Figure 2:**
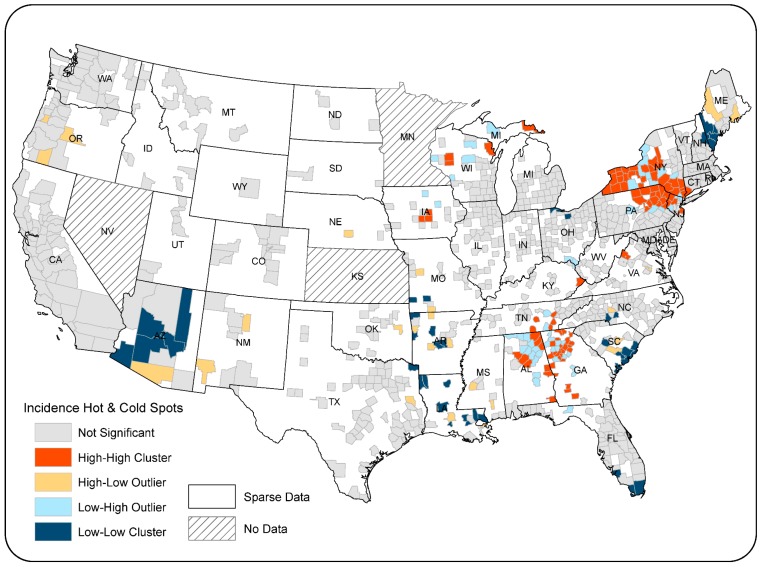
Local indicator of spatial autocorrelation (LISA), county-level ovarian cancer incidence 2009–2013, whites.

Analysis of the geographic distribution of ovarian cancer rates across the 987 counties using the global Moran’s I indicates a significantly clustered pattern (Moran’s I index value = 0.0298, *p* < 0.001). Outcomes of LISA analysis identify statistically significant clusters of high and low rates among counties with non-sparse data. Results indicate that localized clusters of high rates (in red) existed in several locations including northern Wisconsin, upstate and southern New York, northeast Pennsylvania, and dispersed counties in Alabama and Georgia (Figure 2). Three large clusters of low rates appeared in the northeastern and southeastern U.S. and Arizona. Several small clusters were mainly scattered in Missouri, Arkansas, and Louisiana. Orange counties represented outliers of high rates in areas where surrounding counties had lower rates. Light-blue counties were outliers of low rates in higher-rate regions. LISA results can vary slightly each time the procedure is run, due to the number of permutations, each of which randomly rearranges neighborhood values to determine whether the observed value for a county (i.e., incidence rate) is random or significantly different. 

### 3.2. Spatial Patterns of Pulp and Paper Facilities

**Figure 3:**
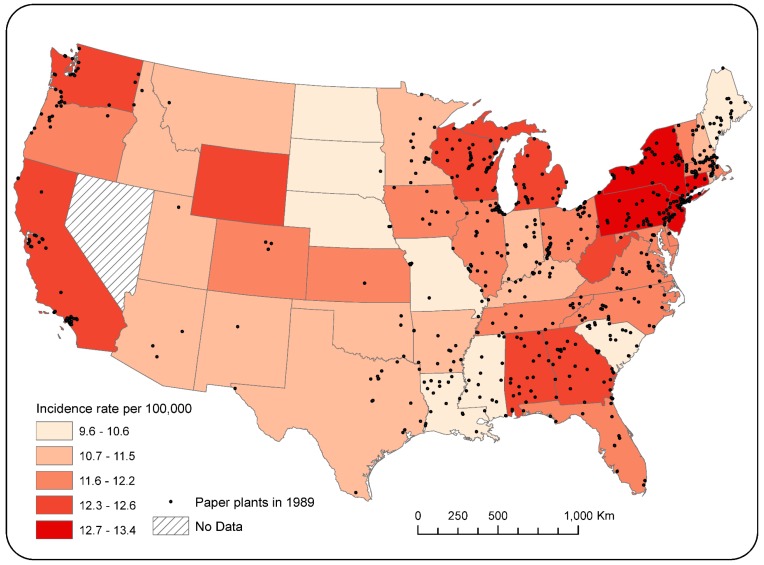
Paper mill locations and state-level ovarian cancer incidence rates by state (2009–2013).

Overlaying state-level ovarian cancer incidence rates with the distribution of pulp and paper plants facilitates visual inspection of the spatial relationship between these variables. Figure 3 shows pulp and paper mill locations for the year 1989 (peak year, *n* = 688), with background shading representing state-level ovarian cancer incidence rates (2009–2013). It is noteworthy that states with the highest ovarian cancer incidence rates (e.g., New York, Pennsylvania, and New Jersey in the Northeast, Wisconsin and Michigan in the Midwest, and Georgia and Alabama in the South) tended to have the largest numbers of paper mills in the late 1980s. 

There has been a notable decline in paper production in the U.S. in the past three decades, with a concomitant decrease in emissions, largely as a result of competition from other countries [44]. In 1988, there were 673 pulp and paper mills in 408 U.S. counties with reported emissions; by 2012, this had dropped to 348 mills in 286 counties. At the state level, in 1989, there were 10 states with more than 20 paper mills; in 2012, there were only two.

**Figure 4:**
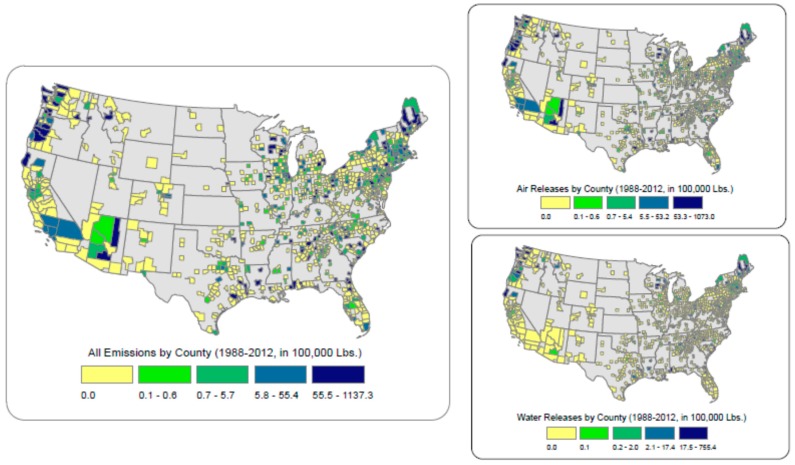
Total onsite emissions and air and water breakdowns from pulp and paper mills, 1988–2012.

Mapping aggregated data on TRI emissions from pulp and paper mills (1988–2012) for U.S. counties enables us to better understand the link with ovarian cancer (Figure 4). Several geographic foci were evident: western Washington and Oregon, Maine, Wisconsin, Louisiana, southwestern Alabama and coastal Georgia, and South Carolina stand out. Air releases reflect overall emissions, but water releases were more restricted. Dark-blue counties on the water releases map indicate that no water effluents were released. Chemicals reported through the TRI process include about 180 known or suspected carcinogens, which EPA refers to as ‘OSHA carcinogens’. The geography of OSHA carcinogen emissions is similar to that of all emissions. However, reported dioxin emissions, measured in grams, are greater in Washington and the southeastern U.S.

**Figure 5:**
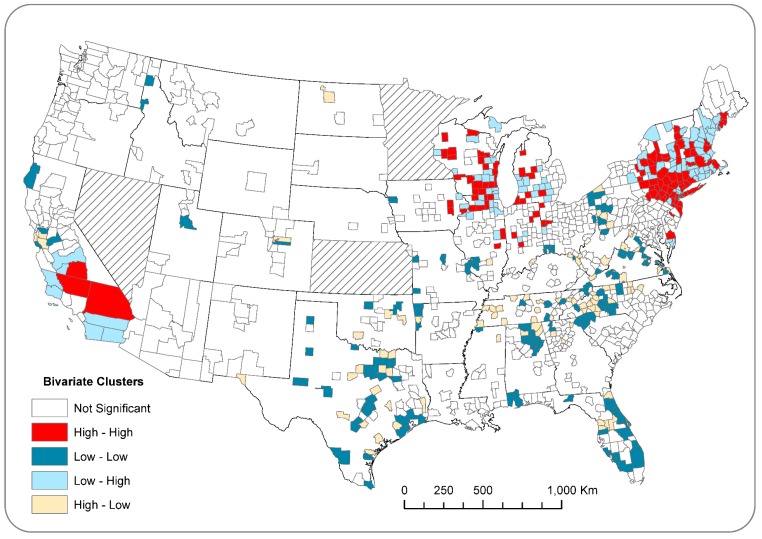
Bivariate LISA map showing clusters of counties with similar ovarian cancer rates and counts of paper mills (i.e., High–High or Low–Low on map).

Results of bivariate LISA analysis indicate an overall significant spatial association between county-level ovarian cancer incidence and the count of paper plants in nearby counties (Moran’s I: 0.0127; *p* < 0.05). The bivariate LISA map (Figure 5) illustrates that 115 counties were labeled as “High–High” (in dark red), which represents clusters of counties that had high incidence rates while being surrounded by counties with high counts of paper mills. Likewise, 139 counties labelled as “Low–Low” (in dark blue) had significantly low incidence rates, whereas nearby counties had low paper mill counts. Clusters of “High–High” and “Low–Low” collectively indicate a positive spatial association between ovarian cancer incidence rates and paper mill counts.

### 3.3. Impacts of Air and Water Emissions on Ovarian Cancer

**Figure 6:**
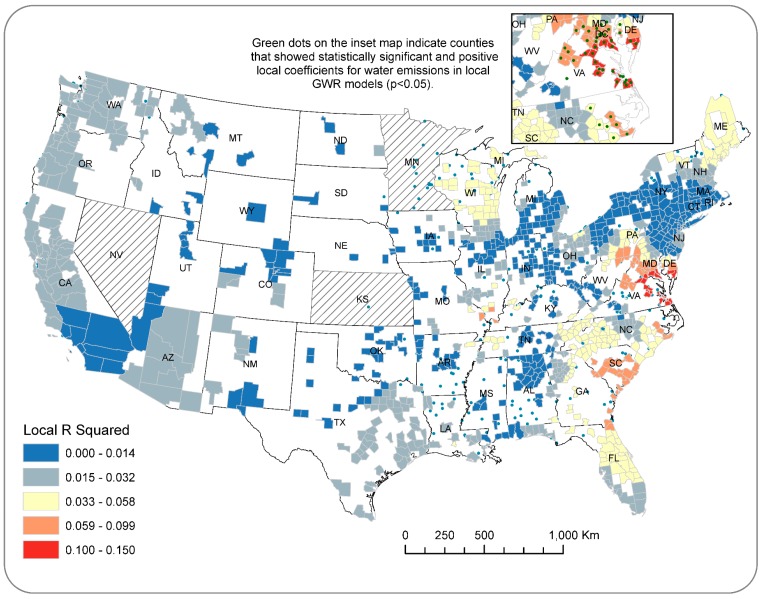
Results of local regression parameters from geographically weighted regression (GWR) models for All Emissions showing spatially varying explanatory power (i.e., local R squared values) for the county-level data.

Conversely, results of the regression analyses for the county-level data did not show significant regression coefficients for the two measures of paper mill emissions in all three OLS models (Table 2). Adjusted R-squared values ranged from 0.002 to 0.005, indicating that paper mill wastes explained less than one percent of the variations in ovarian cancer incidence. However, results of the GWR models dramatically improved the explanatory power of the regression analysis. For “All Emissions”, GWR models overall explained nearly five percent of county-level variation in ovarian incidence rates and the local parameters of the adjusted R squared statistic were between a range of 0.059 and 0.15 for counties along the east coast including Maine, Pennsylvania, Maryland, Delaware, Virginia, North Carolina, South Carolina, and Florida (Figure 6). Moreover, water emissions showed statistically significant and positive local regression coefficients for 48 counties located in the aforementioned regions (illustrated in the inset map in Figure 6), which confirms the finding from the state-level analysis of the relationship between TRI pollution and ovarian cancer incidence.

Among the three OLS models, a statistically significant positive association was identified between surface water emission and ovarian cancer incidence rates for the “All Emissions” model. Among the three OLS models, the best-performing model (i.e., “All Emissions”) explained less than seven percent of the variation of state-level ovarian cancer rates. Significant positive Moran’s I values were observed among the residuals for all three OLS models, warranting the further use of spatial regression methods. Differently from the OLS models, spatially lagged values of the dependent variable, named “Lagged incidence”, were added as an additional independent variable and showed a statistically significant and positive impact on ovarian cancer rate, indicating the rationale of mitigating spatial autocorrelation among geographically adjacent counties. Surface water remained statistically significant in the spatial lag models, although at slightly lower magnitude than the OLS models. Results of the spatial lag models improved the prediction power of the regression analyses indicated by significantly larger adjusted R squared values and lower Akaike info criterion (AICc) statistics.

## 4. Discussion

This is the first report to investigate the spatial patterns of ovarian cancer rates in the U.S. in relation to pulp and paper emissions using advanced geospatial techniques and national datasets at state and county levels. A comparison of the spatial distributions of ovarian cancer and paper mills indicates that areas with a high level of ovarian cancer incidence rates are also more likely to have had large numbers of pulp/paper manufacturing facilities in the late 1980s. Global Moran’s I analysis suggests that ovarian cancer incidence had an overall clustered pattern, whereas local clusters of high rates of incidence were detected in Wisconsin, New York/Pennsylvania, Alabama/Georgia, and south-central Iowa. Bivariate LISA analysis of county-level data reinforces our observations of a positive spatial association between cancer incidence rates and concentrations of paper mills.

Results of regression analyses for state-level data revealed that the amount of water pollutants was positively related to ovarian cancer incidence in the models for all emissions. In particular, the spatial lag models significantly improved the predictive power of the regression analysis and manifested that neighboring states tend to have similar levels of ovarian cancer rates. Findings from the state-level data extend prior research that reported a correlation between ovarian cancer rates in the U.S. and pulp and paper manufacturing [27]. Local models from GWR analysis provide supportive evidence for a significant association between ovarian cancer rates and reported water emissions from pulp and paper mills. This finding justifies the rationale of using GWR to explore relationships of cancer incidence and environmental risk factors that may vary across space.

This research has several limitations, due mainly to the incompleteness of data on cancer incidence and pollutant emissions. For example, the five-year timespan of incidence data (2009–2013) provided by State Cancer Profiles contain only 31 percent of all counties in the U.S. (i.e., 987 counties). Surveillance, Epidemiology, and End Results (SEER) program registries do not exist in most of the states with high pulp and paper production. One option for additional research is to obtain better geographic coverage directly from CDC’s Research Data Center (RDC); however, at least six of the 48 contiguous states do not provide county-level data to the RDC. Future research to clarify this discrepancy should focus on specific, paper-producing states with long-term cancer registries (e.g., Georgia) and could include local analyses of hot spots, including fate and transport modeling.

TRI data have been used in many studies of public health and exposure [45,46], but these data also have limitations. For example, chemical releases are industry-reported estimates and are subject to biases concerning accuracy and potential underreporting [47]. Indeed, there are financial and political incentives for polluters to minimize the quantity of pollutants reported. Additionally, the release of chemicals does not necessarily indicate exposure in a population or the concentration of chemicals in air or water. The latter requires fate and transport modeling. 

Residential mobility and population migration could be a source of confounding in light of the long latency of ovarian cancer. In the 1950s and 60s, one in five Americans moved annually [48]. This decreased to 14 percent in the 2000s and to 11.6 percent in 2011. American Community Survey five-year (2011–2015) estimates indicate that 5.5% of the U.S. population aged 1 year and older migrated across county or state boundaries [49]. Therefore, accounting for population migration in future research could potentially shed new light on the relationship between ovarian cancer and TRI toxic emissions.

Most importantly, we emphasize that this is an ecological study in which measurements were made at the levels of states and counties, not the individual. Thus, one cannot conclude from these findings that exposure to toxic emissions from pulp and paper mills is associated with the risk of ovarian cancer at the individual level. However, several studies at the individual level, for example, Inoue-Choi et al. (2015) [50] and Langseth and Kjaerheim (2004) [25] suggest that water contamination or pollution from the paper industry may increase the risk of ovarian cancer. One method to pursue this lead in future would be to employ a nested case-control study using stored sera. For example, a recent report from the JANUS cohort in Norway demonstrated that higher calcium levels in serum were significantly associated with an increased risk of ovarian cancer decades later [51]. Since a major class of chemicals involved in pulp and paper manufacturing is the organochlorines, a nested case-control study of organochlorine exposure and ovarian cancer, as has been reported for prostate cancer and for lymphoma, would be especially valuable [52,53].

## 5. Conclusions

Application of advanced geospatial methods to ovarian cancer incidence rates in the U.S. at the state and county levels demonstrates that these rates are significantly correlated with water borne pollutant emissions from the pulp and paper industry. Analytic epidemiologic studies of ovarian cancer in relation to emissions from pulp and paper manufacturing are warranted.

## Figures and Tables

**Table 1:** Results of state-level regression analyses.

	Models	OLS Models			Spatial Lag Models		
Variables		All Emissions (*n* = 48)	Dioxin (*n* = 46)	OSHA Carcinogens (*n* = 46)	All Emissions (*n* = 48)	Dioxin (*n* = 46)	OSHA Carcinogens (*n* = 46)
Intercept		11.5283	11.6374	11.5851	5.96116 ***	5.70606 ***	5.53864 ***
Air emissions		0.00002	−0.00119	0.00027	−0.00002	–0.00031	0.00009
Surface water		0.00172 *	0.00628	0.02813	0.00153 *	0.00476	0.02793
Lagged incidence		n.a.	n.a.	n.a.	0.47707 **	0.50422 ***	0.51517 ***
Adjusted R squared		0.06982	−0.02065	0.00485	0.28249	0.2348	0.2658
Akaike info criterion (AICc)		122.754	123.37	122.206	117.442	117.638	115.926
Moran’s I diagnosis of residuals		0.2515 **	0.2877 **	0.3044 ***	0.01048	0.03608	0.04585

* Significance level of 0.05; ** Significance level of 0.01; *** Significance level of 0.001.

**Table 2:** Results of OLS regression for county-level data (*n* = 987).

	Models	OLS Models (*n* = 987)			GWR (*n* = 987)		
Variables		All Emissions	Dioxin	OSHA Carcinogens	All Emissions	Dioxin	OSHA Carcinogens
Intercept		12.6906 ***	12.6549 ***	12.6608 ***	12.7146 ***	12.7280	12.6967
Air emissions		−0.00204 *	−0.03467 *	−0.00715	−0.00201	−0.0387	0.000
Surface water		−0.00187	0.01119	−0.1672	−0.0413	0.0028	0.00002
Adjusted R squared		0.00486	0.00216	0.00359	0.0466	0.0085	0.0412
Akaike info criterion (AICc)		4856.083	4856.72	4855.31	4843.762	4854.897	4849.515

* Significance level of 0.05; ** Significance level of 0.01; *** Significance level of 0.001.

## Data Availability

Data on ovarian cancer incidence are publicly available at the websites of the National Cancer Institute and the Centers for Disease Control (CDC), and data on TRI emissions can be assessed from the website of the U.S. Environmental Protection Agency’s Toxic Release Inventory (EPA TRI). The datasets generated and analyzed during the current study are available from the corresponding author upon request.

## References

[1] American Cancer Society (2017). Cancer Facts and Figures 2017.

[2] Tokuoka S., Kawai K., Shimizu Y., Inai K., Ohe K., Fujikura T., Kato H. (1987). Malignant and benign ovarian neoplasms among atomic bomb survivors, Hiroshima and Nagasaki, 1950-80. J. Natl. Cancer Inst..

[3] Hanna L., Adams M. (2006). Prevention of ovarian cancer. Best Pract. Res. Clin. Obstet. Gynaecol..

[4] Hunn J., Rodriguez G.C. (2012). Ovarian cancer: Etiology, risk factors, and epidemiology. Clin. Obstet. Gynecol..

[5] Schildkraut J.M., Alberg A.J., Bandera E.V., Barnholtz-Sloan J., Bondy M., Cote M.L., Funkhouser E., Peters E., Schwartz A.G., Terry P. (2014). A multi-center population-based case–control study of ovarian cancer in African-American women: The African American Cancer Epidemiology Study (AACES). BMC Cancer.

[6] Cramer D.W., Liberman R.F., Titus-Ernstoff L., Welch W.R., Greenberg E.R., Baron J.A., Harlow B.L. (1999). Genital talc exposure and risk of ovarian cancer. Int. J. Cancer.

[7] Gertig D.M., Hunter D.J., Cramer D.W., Colditz G.A., Speizer F.E., Willett W.C., Hankinson S.E. (2000). Prospective study of talc use and ovarian cancer. J. Natl. Cancer Inst..

[8] Hanchette C.L., Schwartz G.G. (1992). Geographic patterns of prostate cancer mortality. Evidence for a protective effect of ultraviolet radiation. Cancer.

[9] Schwartz G.G., Hanchette C.L. (2006). UV, latitude, and spatial trends in prostate cancer mortality: All sunlight is not the same (United States). Cancer Causes Control.

[10] Boscoe F.P., Schymura M.J. (2006). Solar ultraviolet-B exposure and cancer incidence and mortality in the United States, 1993–2002. BMC Cancer.

[11] Garland C.F., Mohr S.B., Gorham E.D., Grant W.B., Garland F.C. (2006). Role of ultraviolet B irradiance and vitamin D in prevention of ovarian cancer. Am. J. Prev. Med..

[12] Grant W.B. (2002). An estimate of premature cancer mortality in the US due to inadequate doses of solar ultraviolet-B radiation. Cancer.

[13] Grant W.B., Garland C.F. (2006). The association of solar ultraviolet B (UVB) with reducing risk of cancer: Multifactorial ecologic analysis of geographic variation in age-adjusted cancer mortality rates. Anticancer Res..

[14] Lefkowitz E.S., Garland C.F. (1994). Sunlight, vitamin D, and ovarian cancer mortality rates in US women. Int. J. Epidemiol..

[15] Cook L.S., Neilson H.K., Lorenzetti D.L., Lee R.C. (2010). A systematic literature review of vitamin D and ovarian cancer. Am. J. Obstet. Gynecol..

[16] Malvezzi M., Carioli G., Rodriguez T., Negri E., la Vecchia C. (2016). Global trends and predictions in ovarian cancer mortality. Ann. Oncol..

[17] Parkin D.M. (1989). Cancers of the breast, endometrium and ovary: Geographic correlations. Eur. J. Cancer Clin. Oncol..

[18] Runnebaum I.B., Stickeler E. (2001). Epidemiological and molecular aspects of ovarian cancer risk. J. Cancer Res. Clin. Oncol..

[19] Torre L.A., Bray F., Siegel R.L., Ferlay J., Lortet-Tieulent J., Jemal A. (2015). Global cancer statistics, 2012. CA Cancer J. Clin..

[20] Coburn S., Bray F., Sherman M., Trabert B. (2017). International patterns and trends in ovarian cancer incidence, overall and by histologic subtype. Int. J. Cancer.

[21] U.S. Cancer Statistics Working Group (2017). United States Cancer Statistics: 1999–2014 Incidence and Mortality Web-Based Report.

[22] García-Pérez J., Lope V., Lopez-Abente G., Gonzalez-Sanchez M., Fernandez-Navarro P. (2015). Ovarian cancer mortality and industrial pollution. Environ. Pollut..

[23] Langseth H., Andersen A. (1999). Cancer incidence among women in the Norwegian pulp and paper industry. Am. J. Ind. Med..

[24] Soskolne C.L., Sieswerda L.E. (2010). Cancer risk associated with pulp and paper mills: A review of occupational and community epidemiology. Chronic Dis. Can..

[25] Langseth H., Kjaerheim K. (2004). Ovarian cancer and occupational exposure among pulp and paper employees in Norway. Scand. J. Work Environ. Health.

[26] Camargo M.C., Stayner L.T., Straif K., Reina M., Al-Alem U., Demers P.A., Landrigan P.J. (2011). Occupational exposure to asbestos and ovarian cancer: A meta-analysis. Environ. Health Perspect..

[27] Schwartz G.G., Sahmoun A.E. (2014). Ovarian cancer incidence in the United States in relation to manufacturing industry. Int. J. Gynecol. Cancer.

[28] Parenteau M.-P., Sawada M.C. (2011). The modifiable areal unit problem (MAUP) in the relationship between exposure to NO_2_ and respiratory health. Int. J. Health Geogr..

[29] Schwartz G.G., Rundquist B.C., Simon I.J., Swartz S.E. (2017). Geographic distributions of motor neuron disease mortality and well water use in U.S. counties. Amyotroph. Lateral Scler. Frontotemporal Degener..

[30] Holt D., Steel D., Tranmer M. (1996). Area homogeneity and the modifiable areal unit problem. Geogr. Syst..

[31] Centers for Disease Control and Prevention, National Cancer Institute (2017). State Cancer Profiles.

[32] Moorman P.G., Palmieri R.T., Akushevich L., Berchuck A., Schildkraut J.M. (2009). Ovarian cancer risk factors in African-American and white women. Am. J. Epidemiol..

[33] Ness R.B., Grisso J.A., Klapper J., Vergona R. (2000). Racial differences in ovarian cancer risk. J. Natl. Med. Assoc..

[34] U.S. Environmental Protection Agency (2017). TRI Explorer.

[35] Karoutsou E., Karoutsos P., Karoutsos D. (2017). Endocrine Disruptors and Carcinogenesis. Arch. Cancer Res..

[36] Anselin L. (1999). Interactive techniques and exploratory spatial data analysis. Geographical Information Systems: Principles, Techniques, Management and Applications.

[37] Kanaroglou P., Delmelle E. (2016). Spatial Analysis in Health Geography.

[38] Sridharan S., Tunstall H., Lawder R., Mitchell R. (2007). An exploratory spatial data analysis approach to understanding the relationship between deprivation and mortality in Scotland. Soc. Sci. Med..

[39] Anselin L. (2009). Spatial Regression. The SAGE Handbook of Spatial Analysis.

[40] Fotheringham A.S., Brunsdon C., Charlton M. (2003). Geographically Weighted Regression: The Analysis of Spatially Varying Relationships.

[41] Kirby R.S., Delmelle E., Eberth J.M. (2017). Advances in spatial epidemiology and geographic information systems. Ann. Epidemiol..

[42] Rezaeian M., Dunn G., Leger S.S., Appleby L. (2007). Geographical epidemiology, spatial analysis and geographical information systems: A multidisciplinary glossary. J. Epidemiol. Community Health.

[43] Brewer C.A. (2006). Basic mapping principles for visualizing cancer data using Geographic Information Systems (GIS). Am. J. Prev. Med..

[44] McLaughlin P. (2015). Where the Paper Industry went. Bangor Daily News.

[45] Choi H.S., Shim Y.K., Kaye W.E., Ryan P.B. (2006). Potential residential exposure to toxics release inventory chemicals during pregnancy and childhood brain cancer. Environ. Health Perspect..

[46] Dickerson A.S., Rahbar M.H., Han I., Bakian A.V., Bilder D.A., Harrington R.A., Pettygrove S., Durkin M., Kirby R.S., Wingate M.S. (2015). Autism spectrum disorder prevalence and proximity to industrial facilities releasing arsenic, lead or mercury. Sci. Total Environ..

[47] Neumann C.M. (1998). Improving the U.S. EPA Toxic Release Inventory database for environmental health research. J. Toxicol. Environ. Health B Crit. Rev..

[48] Rowntree L., Lewis M., Price M., Wyckoff W. (2017). Diversity amid Globalization: World Regions, Environment, Development.

[49] U.S. Census Bureau, American Community Survey (2017). Geographic Mobility in the Past Year by Age for Current Residence in the United States (B07001).

[50] Inoue-Choi M., Jones R.R., Anderson K.E., Cantor K.P., Cerhan J.R., Krasner S., Robien K., Weyer P.J., Ward M.H. (2015). Nitrate and nitrite ingestion and risk of ovarian cancer among postmenopausal women in Iowa. Int. J. Cancer.

[51] Schwartz G.G., Tretli S., Vos L., Robsahm T.E. (2017). Prediagnostic serum calcium and albumin and ovarian cancer: A nested case-control study in the Norwegian Janus Serum Bank Cohort. Cancer Epidemiol..

[52] Koutros S., Langseth H., Grimsrud T.K., Barr D.B., Vermeulen R., Portengen L., Wacholder S., Freeman L.E., Blair A., Hayes R.B. (2015). Prediagnostic serum organochlorine concentrations and metastatic prostate cancer: A Nested Case-Control Study in the Norwegian Janus Serum Bank Cohort. Environ. Health Perspect..

[53] Laden F., Bertrand K.A., Altshul L., Aster J.C., Korrick S.A., Sagiv S.K. (2010). Plasma organochlorine levels and risk of non-Hodgkin lymphoma in the Nurses' Health Study. Cancer Epidemiol. Biomark. Prev..

